# Shikonin Mediates Apoptosis through G Protein-Coupled Estrogen Receptor of Ovarian Cancer Cells

**DOI:** 10.1155/2022/6517732

**Published:** 2022-10-05

**Authors:** Xinyue Liu, Yao Yang, Xuefei Tang, Li Guo, Xinhui Tang, Ting Zhu, Tiannan Zhao, Weina Zhang, Ping Zhang

**Affiliations:** ^1^Department of Gynecology, Qingdao Municipal Hospital, Qingdao 266011, China; ^2^Graduate School, Qingdao University, Qingdao 266011, China

## Abstract

This study was intended to establish the predictive target of Shikonin (SK) against ovarian cancer using network pharmacology and to clarify the potential mechanism of SK in promoting apoptosis in ovarian cancer. Cell Counting Kit-8 assay, plate clone assays, LDH assay, flow cytometric analysis of Annexin V-fluorescein isothiocyanate/propidium iodide staining, and western blotting were used to assess the effect of SK on apoptosis of ovarian cancer cell lines (SKOV3 and A2780). Pharmacodynamic targets were used to predict the targets of SK and ovarian cancer. Gene Ontology (GO) enrichment analysis and Kyoto Encyclopedia of Gene and Genome (KEGG) pathway enrichment analyses were used to analyze the biological functions and signal pathways of these targets. SK promoted apoptosis in ovarian epithelioid adenocarcinoma cells. SK-ovarian cancer pharmacodynamic target analysis screened 17 related genes. GO and KEGG analyses showed that SK affected the estrogen signaling pathway. SK inhibited the expression of GPER in SKOV3 and A2780 cells and downregulated the expression of EGFR, p-EGFR, PI3K, and *p*-AKT in a concentration-dependent manner. The apoptosis-promoting effect of SK was enhanced by GPER-specific agonist G1 and inhibited by the specific inhibitor G15. The expression of EGFR, *p*-EGFR, PI3K, and *p*-AKT was decreased by G1 and reversed by G15. SK also inhibited tumor growth in the SKOV3 xenograft model, and it acted synergistically with G1. However, the effect can be attenuated by G15 in vivo. In summary, SK may affect the apoptosis of ovarian cancer cells through GPER/EGFR/PI3K/AKT, and GPER may be a key target of SK in ovarian cancer cell apoptosis.

## 1. Introduction

Ovarian cancer is one of the most common malignancies of the female reproductive system, and it has one of the highest mortality rates. Because early pathological changes are difficult to detect, and the disease progresses rapidly, ovarian cancer has already become one of the most serious threats to the health of women worldwide [1]. Although standard therapy for advanced ovarian cancer, such as cytoreductive surgery, followed by chemotherapy, has made great progress, the side effects of chemotherapy are inevitable [2]. Therefore, we hope to find some traditional Chinese medicines for the prevention and treatment of ovarian cancer and to explore its mechanism of action for more effective treatment.

SK is one of the main active ingredients isolated from the dried roots of *Lithospermum erythrorhizon*. It has various biological functions, such as antitumor, anti-inflammatory, antiestrogen, antibacterial, and antiviral effects [3]. In recent years, research has proven that SK has a significant antitumor effect, including breast cancer, cervical cancer, lung cancer, and digestive system tumors. It has been reported that the antitumor mechanisms of SK involve inhibition of proliferation, arrest of the cell cycle, and regulation of related protein expression, among others [4]. However, the specific mechanism of SK in ovarian cancer and the guidance for clinical application require further study.

Network pharmacology is an emerging drug design discipline that can improve the efficacy of drugs and the success rate of clinical trials and reduce the costs of drug development by predicting drug active ingredients, drug efficacy targets, and signal pathways [5]. In this study, the network pharmacological analysis identified common targets for SK and ovarian cancer, and KEGG and GO analyses revealed that these common targets were enriched in pathways associated with the estrogen signaling pathway, which was mainly located in the membrane region for a series of biological processes. A membrane estrogen receptor called G protein-coupled estrogen receptor (GPER) mediates rapid nongenomic effects and plays an important role in the occurrence and development of estrogen-related tumors such as breast, ovarian, cervical, and endometrial cancers [6]. The phosphatidylinositide 3-kinase (PI3K)/AKT signaling pathway is one of the essential routes in the molecular mechanism of proliferation and apoptosis regulation in human cells. Estrogenic ligand signaling through GPER is propagated by transactivation of the plasma membrane tyrosine kinase receptor epidermal growth factor receptor (EGFR), which activates the PI3K/*p*-AKT signaling pathway [7]. Since activation of PI3K/*p*-Akt signal transduction is the main driver of cell growth, we hypothesized that SK may inhibit the transduction of EGFR/PI3K/AKT pathway through the estrogen signaling pathway, causing the inhibition of the growth of ovarian cancer cells.

In this study, we demonstrated that SK promotes the apoptosis of ovarian cancer in vitro and in vivo, and its possible molecular mechanism by inhibiting the GPER-mediated EGFR/PI3K/AKT signaling pathway.

## 2. Material and Methods

### 2.1. Chemicals and Reagents

Shikonin (SK, purity ≥98.0% by HPLC, molecular weight: 288.30) was purchased from Sigma-Aldrich (Beijing, China). Five milligrams of SK was dissolved in 5 ml dimethyl sulfoxide (DMSO, D2650, Sigma, USA) to make a 3.5 mM stock solution, which was then added to the medium at the indicated concentrations. GPER-specific agonists G-1 and G-15 were obtained from MCE (Shanghai, China). 5 mg of G1 and 1 mg of G15 were, respectively, dissolved in 1.2128 mL and 2.7010 mL of DMSO and were dissolved in 10 mM G1 and 1 mM G15 stock solution by ultrasonic shock for subsequent experiments. The Cell Counting Kit-8 (CCK-8) reagent (K1018) was purchased from APExBIO (Houston, USA), and the Lactate Dehydrogenase (LDH) Kit was purchased from Solarbio Science and Technology Co., Ltd (Beijing, China), while the Annexin V-FITC/PI staining kit was obtained from Absin Bioscience Inc. (abs50001; Beijing, China). RIPA lysis buffer (P0013b; Beyotime, China) and protein phosphatase inhibitor (Solarbio, Beijing, China) were purchased for use. Additionally, Matrigel glue (BD Biosciences, NJ, USA), BCA protein analysis kit (Absin, Beijing, China), and ECL reagent (Millipore, Massachusetts, USA) were obtained in the present study. The following antibodies were used: anti-GPER (PY2737S; Abcam, China), anti-PI3K (20584-1-AP; Proteintech, China), phospho-Akt (Ser473) antibody (66444-1-lg; Proteintech, China), anti-Bax (2774; CST, USA), and Bcl2 (3498; CST, USA). Fluorescein-conjugated goat anti-rabbit IgG (*H* + *L*) (ab6721; Abcam, USA) and DAPI were purchased from Solarbio (Beijing, China).

### 2.2. Cell Culture

Human ovarian adenocarcinoma SKOV3 cells and A2780 cells were purchased from the Chinese Academy of Sciences (Shanghai) Cell Bank and maintained in Dulbecco's modified Eagle's medium (DMEM) (HyClone, USA) supplemented with 10% fetal bovine serum (FBS, GIBCO, USA) and 1% penicillin-streptomycin (HyClone, UT, USA). All cells were maintained at 37°C in a humidified atmosphere of 95% air and 5% CO_2_. SKOV3 cells were subcultured every 2-3 days, and A2780 cells were subcultured every 1-2 days. Phenol red-free DMEM (10% FBS, 1% penicillin-streptomycin; GIBCO, USA) medium was applied on the fourth day before the experiment to improve the sensitivity of cells to estrogenic substances and reduce the interference of estrogen in the cells on experimental results. The cells were selected for the experiment in the logarithmic growth phase, and the experiment was strictly performed according to the laboratory's aseptic operation principle.

### 2.3. Cell Counting Kit 8 (CCK8) Test

The cytotoxicity of SK on SKOV3 and A2780 cells was determined using the CCK-8 assay. SKOV3 or A2780 cells were seeded in 96-well plates at a density of 5 × 10^3 cells per well in the growth medium. After 12 h, the cells were treated with different concentrations of SK at different time, and then, 10 *µ*L of CCK-8 reagent was added to each well. After incubation at 37°C for 2 h, the optical density (OD) was measured at 450 nm, and the relative cell viability and cell viability were calculated. Five replicate wells were used for each experimental condition.

### 2.4. Determination of Lactate Dehydrogenase (LDH)

After different concentrations of SK treated SKOV3 or A2780 cells for 24 h, as described by the manufacturer, the LDH kit was utilized to measure the LDH release level of damaged cells to evaluate cytotoxicity.

### 2.5. Plate Cloning

SKOV3 or A2780 cells were cultured at a density of 500 cells per well in 6-well plates and gently shaken to evenly disperse the cells. After 12 h, SKOV3 or A2780 cells were treated with SK and incubated in a cell culture incubator at 37°C with 5% CO_2_. After 2 weeks, the cells were washed 3 times with Phosphate buffered saline (PBS, Solarbio, Beijing, China). Subsequently, the cells were fixed with 4% paraformaldehyde for 15 min and stained with crystal violet for 10 min. The staining solution was then washed with PBS. The six-well plate was inverted, covering the transparent sheet with a grid, and the clones were directly counted with the naked eye: clone formation rate = (number of clones/number of cells inoculated) × 100%.

### 2.6. Annexin V-FITC/PI Assay

The cells were inoculated into 6-well plates at a density of 1 × 10^5^ cells per well. After 12 h, the cells were treated with SK for 24 h. Then, the cells were collected and resuscitated with precooled 1 × PBS and washed with 300 *µ*L of 1 × binding buffer, and 5 *µ*L Annexin V-FITC was added and incubated in the dark at 20°C for 15 min. Before testing, 5 *µ*L PI and 200 *µ*L 1 × binding buffers were added. Finally, the samples were analyzed using an Accuri C6 flow cytometer (BD Biosciences, CA, USA). Data were analyzed using FlowJo VX.0.7 software. Both early apoptotic (Annexin V+ and PI−) and late apoptotic (Annexin V+ and PI+) cells were detected.

### 2.7. Composition and Pharmacokinetic ADME Prediction

All compositions of Zicao were acquired from the Traditional Chinese Medicines for Systems Pharmacology Database and Analysis Platform (TCMSP, available online: https://tcmspw.com/tcmsp.php). The TCMSP database was used to select all compositions based on absorption, distribution, metabolism, and excretion (ADME) criteria. In our study, the active ingredients with good oral bioavailability (OB) and drugd properties (DL) (OB ≥ 30% and DL ≥ 0.18) [8] were screened out, and gene annotation of the TCMCP targets was conducted on the UniProt website (https://www.uniprot.org/).

### 2.8. Prediction Targets of Compounds

The GeneCards database (https://www.genecards.org/), OMIM database (https://www.omim.org/), TTD database (https://db.idrblab.net/ttd/), PhramGkb database (https://www.pharmgkb.org/), and Drug Bank database (https://go.drugbank.com/) were searched for disease targets related to ovarian cancer and screened from GeneCards with a correlation score greater than 1 target, to remove duplicate genes obtained from the above five gene banks. Using *R* software to draw the Venn diagram, the union of ovarian cancer-related genes and the intersection of the corresponding targets of Zicao and ovarian cancer-related targets were obtained, which is the pharmacological target of Zicao for the treatment of ovarian cancer.

### 2.9. Protein-Protein Interaction (PPI) Network

The active ingredients of Zicao were mapped to the related targets of ovarian cancer, and the process of treating ovarian cancer was visually analyzed using Cytoscape v3.6.1 software, and the interaction network of effective target proteins in the treatment of ovarian cancer was constructed. Efficacy targets obtained by Venn diagram were imported into the STRING database (https://string-db.org/) with the species limited to “*Homo sapiens*” and a confidence score >0.50, to obtain a PPI network.

### 2.10. Enrichment Analysis

On the basis of R-Package cluster Profiler, the target protein was analyzed by GO enrichment analysis and KEGG pathway enrichment analysis. *P* < 0.001 indicated statistical significance. The KEGG database was established in 1995 by the Kanehisa Laboratory of the Bioinformatics Center of Kyoto University, and it is usually used in network pharmacology to annotate the pathway of target genes [9, 10]. Sorting was completed according to the size of the *P*-value, drawing the top 10 biological process (BP), cell component (CC), and molecular function (MF) GO analysis diagrams. The KEGG signal pathway diagram was drawn similarly. The genomes related to the optimal pathways screened by KEGG from MSigDB were collected, and the ggcor package was used to analyze their correlation with these efficacy target genes.

### 2.11. Western Blot Analysis

SKOV3 or A2780 cells were washed twice with precooled PBS and placed in RIPA buffer supplemented with 1% protein phosphatase. The total protein extract was placed in different centrifuge tubes at 4°C and centrifuged at 12000 rpm for 10 min. Then, the protein concentration was determined using the BCA protein analysis reagent and boiling the cell solutions for 5 min with the protein extract. After the protein was separated by SDS-PAGE (10%), the protein was transferred to a PVDF membrane. At room temperature, the membranes were sealed with 5% nonfat dry milk in Tris-buffered saline with 0.05% Tween-20 (TBST) buffer for 2 h. Then, the target membrane and specific primary antibody were incubated overnight at 4°C and washed with TBST three times, and then diluted HRP coupled with secondary antibody was added and incubated at room temperature for 2 h. Finally, an ECL detection kit was used to detect immune complexes. Measurement data were obtained from three different experiments. The strength of each band was measured using the ImageJ software. GAPDH or *β*-actin was used as the control.

### 2.12. Animal Model

6-week-old BALB/*c* nude female mice were obtained from the Model Animal Research Center of Qingdao University (Qingdao, China) and maintained under specific pathogen-free conditions at Qingdao University. The concentration of SKOV3 cells was adjusted to a single cell suspension of 1 × 10^6/ml, and 0.2 ml of the cell suspension was, respectively, inoculated into 20 female health nude mice under the right axillary subcutaneous of the mice. Then, they were randomly divided into four groups: (1) control group (corn oil, *n* = 5), (2) SK group (SK, 10 mg/kg, *n* = 5), (3) *G*1 + SK group (G1, 1 *μ*g/mice daily; SK, 10 mg/kg, *n* = 5), and (4) G15 + SK group(G15, 5 *μ*g/mice daily; SK, 10 mg/kg, *n* = 5) [11]. 24 h after nude mice were inoculated with cancer cells, SK, G-1, and G-15 were diluted in corn oil and intraperitoneally injected for 22 days, and the nude mice were sacrificed under intravenous anesthesia and then measured the tumor mass. Review was performed and approval provided for all animal experiments by the Research Ethics Committee of Qingdao Municipal Hospital (Ethics Approval No. 092). All experiments in this study were performed under the supervision and guidance of the Ethics Committee of Qingdao Municipal Hospital and in strict accordance with the ARRIVE guidelines for animal experiments.

### 2.13. Statistical Analysis

All the experimental results listed in the article are expressed as the mean ± SD, which represents the data of at least three independent repeated experiments. All data analyses were performed using the statistical software GraphPad Prism 7.0 (LaJolla, California, USA). The differences between the two groups were tested using a *t*-test. Multiple groups were compared using a one-way analysis of variance. Results were considered statistically significant if *P* < 0.05.

## 3. Results

### 3.1. The Cytotoxicity of SK in SKOV3 and a2780 Cells Are Dose- and Time-Dependent

**Figure 1:**
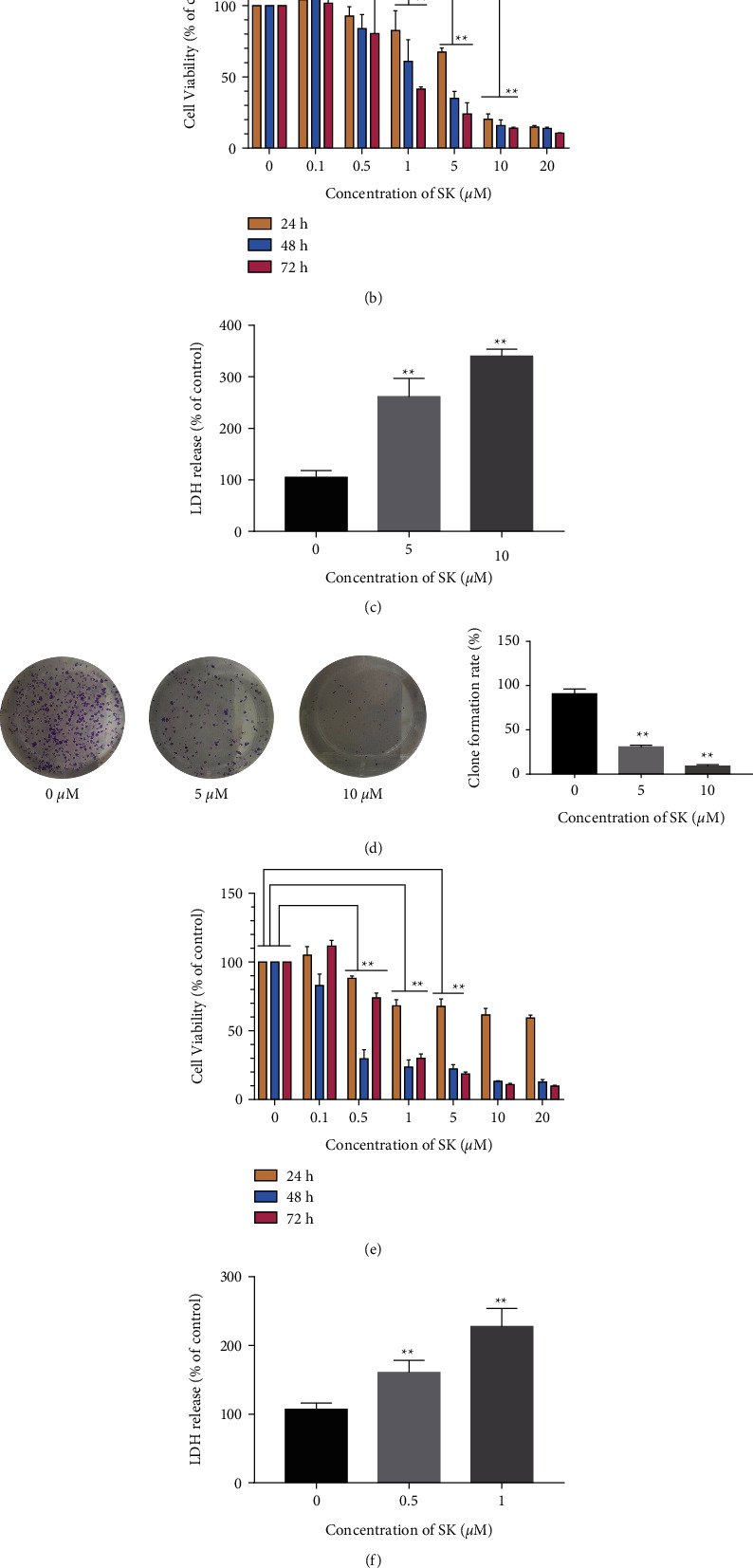
Effects of different concentrations of SK on the proliferation of SKOV3 and A2780 cells. (a) 2D and 3D chemical structure of SK. (b) Cell viability of SKOV3 cells treated with different concentrations of SK for different times. (c) LDH production was measured using an LDH release assay kit in SKOV3 cells treated with 5 and 10 *μ*M SK for 24 h. (d) Plate clone formation experiments including SKOV3 cells with SK at the indicated concentrations (5 and 10 *μ*M) for two weeks. The number of clones was counted. (e) Cell viability of A2780 cells treated with different concentrations of SK for different times. (f) LDH production was measured using an LDH release assay kit in A2780 cells treated with 0.5 and 1.0 *μ*M SK for 24 h. (g) A2780 cells with SK at the indicated concentrations (0.5 and 1.0 *μ*M) for two weeks. The number of clones was counted. Data was represented as means ± SEM. ^*∗*^*P* < 0.05, ^*∗∗*^*P* < 0.01 vs. control group were considered as statistically significantly.

The 2D and 3D chemical structures of SK are shown in Figure 1(a). First, we evaluated its cytotoxicity to SKOV3 cells using the CCK-8 assay. As shown in Figure 1(b), when the concentration is 0.1–0.5 *μ*M, the cytotoxicity of SK against SKOV3 cells was not obvious. At a concentration of 1 *μ*M, the cytotoxicity of SK increased significantly in a dose-andtime-dependent manner. We calculated that the concentration IC50 for 24 h was 8.6 *μ*M. LDH leakage is an index for detecting necrosis and necroptotic program cell death due to damage cytomembrane. The LDH assays indicated that 5 and 10 *μ*M SK treatments for 24 h increased the damage of SKOV3 cells in a dose-dependent way (Figure 1(c)). The plate cloning experiment showed that, compared with the control group, 5 and 10 *μ*M SK significantly inhibited the growth of SKOV3 cells (Figure 1(d)). A similar result was observed in A2780 cells. As shown in Figure 1(e), when the concentration increased from 0.5 to 5 *μ*M for 24 h, the cytotoxicity of shikonin was significantly increased, and cells almost lost their viability at 10 *μ*M and 20 *μ*M. The concentration IC50 for 24 h was 0.56 *μ*M. The LDH assays indicated that 0.5 and 1.0 *μ*M SK treatment for 24 h increased the damage of A2780 cells in a dose-dependent way (Figure 1(f)). The plate cloning experiment showed that 0.5 and 1.0 *μ*M SK also inhibited the growth of A2780 cells (Figure 1(g)).

### 3.2. SK Induces Cell Apoptosis in SKOV3 and a2780 Cells

**Figure 2:**
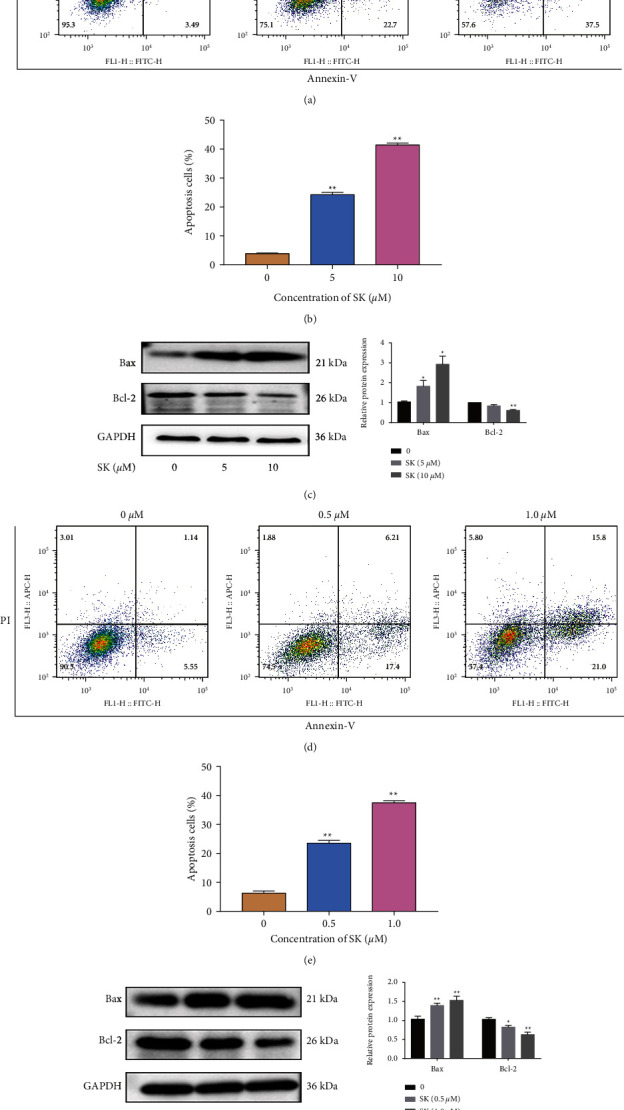
. Effects of SK on the apoptosis of SKOV3 and A2780 cells. (a) Representative dot‑plots from cytometrically illustrating apoptotic status in SKOV3 cells with 5 and 10 *μ*M SK for 24 h. (b) Statistical analysis of apoptotic (percentage of cells in Q2 and Q3) SKOV3 cells. (c) The expression and quantitatively analysis of Bax and Bcl-2 proteins in SKOV3 cells treated with 5 and 10 *μ*M SK for 24 h. (d) Representative dot‑plots from cytometrically illustrating apoptotic status in A2780 cells with 0.5 and 1.0 *μ*M SK for 24 h. (e) Statistical analysis of apoptotic (percentage of cells in Q2 and Q3) A2780 cells. (f) The expression and quantitatively analysis of Bax and Bcl-2 proteins in A2780 cells treated with 0.5 and 1.0 *μ*M SK for 24 h. Images are representative of three independent experiments. Data are mean ± S.E.M. from three independent experiments. ^*∗*^*P* < 0.05, ^*∗∗*^*P* < 0.01 vs. control group were considered as statistically significantly.

To further evaluate whether the cytotoxicity of SK is related to apoptosis, we performed flow cytometry to determine the apoptotic rate and western blotting to analyze the apoptosis-related protein expression of Bax and Bcl-2. In SKOV3 cells, the apoptotic rate was 3.84% in the blank control group, 24.17% in the 5 *μ*M SK group, and 40.79% in the 10 *μ*M SK group (Figures 2(a) and 2(b)). The expression levels of the proapoptotic protein Bax were significantly increased following treatment with 5 and 10 *μ*M SK for 24 h compared to those in the control. On the contrary, the expression levels of the antiapoptotic protein Bcl-2 were decreased (Figure 2(c)). In A2780 cells, the apoptotic rates were 6.69% in the blank control group, 23.61% in the 0.5 *μ*M group, and 36.8% in the 1 *μ*M group (Figures 2(d) and 2(e)). The protein expression of Bax and Bcl-2 was similar to that in SKOV3 cells (Figure 2(f)).

### 3.3. Shikonin Performs Pharmacological Action on the Cell Membrane of Ovarian Cancer through Estrogen Signaling Pathway

**Figure 3:**
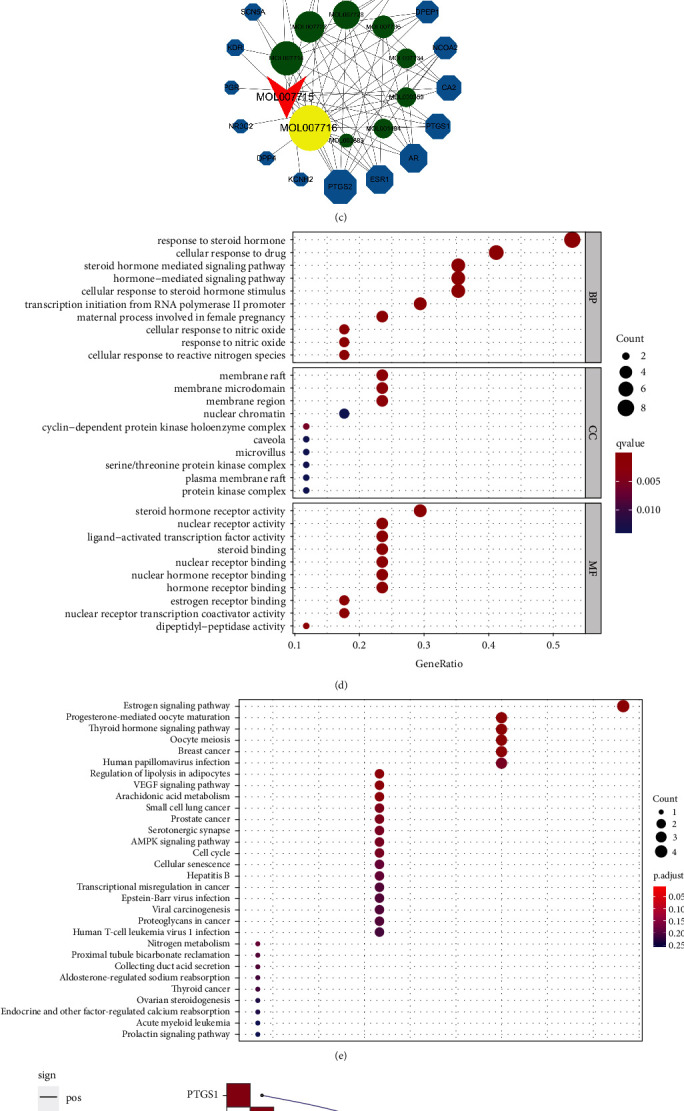
Network pharmacology for investigating pharmacological mechanisms of Zicao acting on ovarian cancer. (a) The Venn diagram of 17 target genes of Zicao. (b) Protein-protein interaction network of the anti-ovarian cancer targets of Zicao. (c) The herb-compoundtarget-ovarian network. (d) GO analysis of common target genes. (e) KEGG analysis of common target genes. (f) Correlation between the main pathway and common target genes.

We used the two ADME-related models, OB and DL, to screen Zicao with related targets, and 12 effective ingredients (OB ≥ 30% and DL ≥ 0.18) were included in the study (Table 1). We obtained ovarian cancer-related targets from GeneCards, OMIM, TTD, PharmGKB, and Drug Bank and predicted the targets of effective ingredients in Zicao from the TCMSP database. After cross-analysis, 17 common targets related to drug diseases were identified (Figure 3(a)). The 17 target genes were subjected to STRING to obtain PPI data (Figure 3(b)). The data sheet obtained from the PPI network was used to input the network pharmacology diagram into Cytoscape 3.6.1 (Figure 3(c)). The green node represents the active ingredient of Zicao, the blue node represents the target of ovarian cancer, and the size represents the degree value of the effective ingredients of Zicao and the disease target. Among them, the most effective ingredients with a higher degree of Zicao are acetylshikonin, 5,8-dihydroxy-2-[(1R)-1-hydroxy-4-methylpent-3-en-1-yl]aphthalene-1,4-dione, isoarnebin 4, and 1-methoxyacetylshikonin. It is worth noting that 5,8-dihydroxy-2-[(1R)-1-hydroxy-4-methylpent-3-en-1-yl]aphthalene-1,4-dione is the chemical name of SK. Next, we analyzed the GO and KEGG results for the common targets of Zicao and ovarian cancer. The GO analysis of the pharmacological targets of Zicao for the treatment of ovarian cancer showed that the main BP was response to steroid hormone, steroid hormone-mediated signaling pathway, and hormone-mediated signaling pathway. The significant MF is steroid hormone receptor activity, which is mainly located in the cell membrane to carry out a series of biological processes (Figure 3(d)). KEGG analysis results and pathway diagrams showed that Zicao mainly affected the occurrence and development of ovarian cancer through the estrogen signaling pathway (Figure 3(e)). Figure 2(f) more intuitively shows the correlation between the estrogen membrane receptor (GPER) signaling pathway and the 17 common genes.

### 3.4. SK Regulates the GPER/EGFR/PI3K/AKT Signaling Pathway

**Figure 4:**
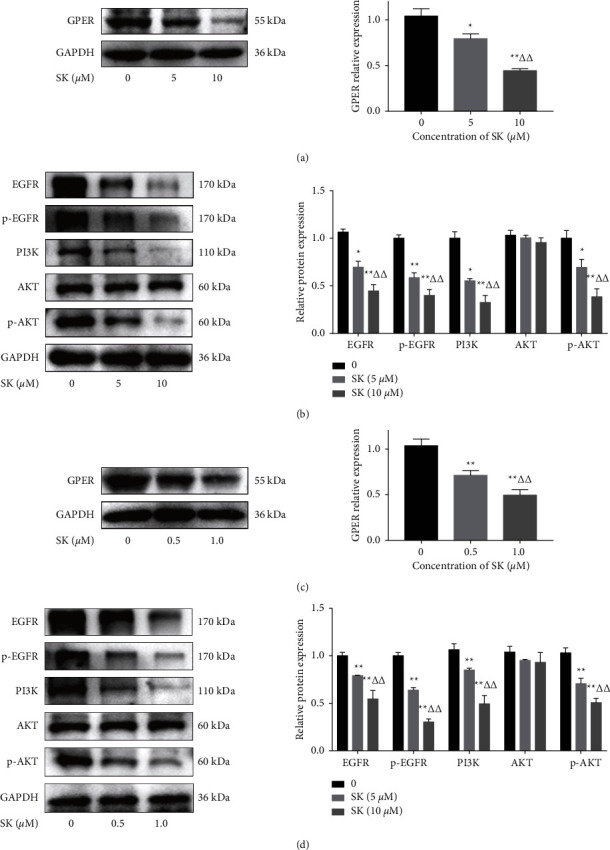
. Western blot analysis of estrogen membrane receptor (GPER) signaling pathway proteins. (a, b) Western blot result of proteins related to GPER/EGFR/PI3K/AKT pathway of SKOV3 cells treated with 5 and 10 *μ*M SK for 24 h. (c, d) Western blot results for proteins related to the GPER/EGFR/PI3K/ AKT pathway in A2780 cells treated with 0.5 and 1.0 *μ*M SK for 24 h. The results are means of three independent replicates ± S.D.^*∗*^*P* < 0.05 or ^*∗∗*^*P* < 0.01 vs. control group were considered as statistically significantly.

In order to verify whether SK promotes apoptosis of SKOV3 and A2780 cells on the cell membrane through the estrogen signaling pathway, we used western blotting to evaluate the effect of SK on GPER expression. The results showed that, in SKOV3 cells, compared to the control group, 5 and 10 *μ*M SK downregulated the expression of GPER in a dose-dependent manner (Figure 4(a)). Next, we tested the expression of EGFR, p-EGFR, PI3K, AKT, and p-AKT after SK treatment. Image analysis also demonstrated an obvious dose-dependent reduction in EGFR, p-EGFR, PI3K, and p-AKT expression in response to SK treatment; however, there was no significant change in the expression of AKT (Figure 4(b)). In A2780 cells, 0.5 and 1.0 *μ*M SK also downregulated GPER, EGFR, p-EGFR, PI3K, and p-AKT in a dose-dependent manner, and the expression of AKT did not change obviously (Figures 4(c) and 4(d)).

### 3.5. SK Downregulated EGFR/P-EGFR/PI3K/P-AKT Protein Expression Is Realized in GPER Mediated Manner of SKOV3 and a2780 Cells

**Figure 5:**
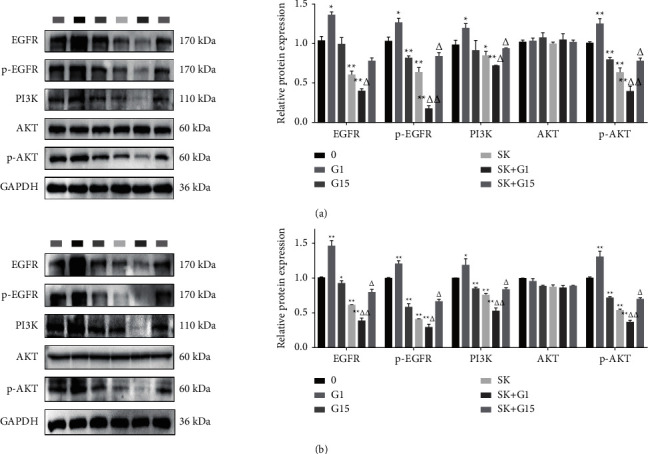
Regulation of EGFR/PI3K/p-AKT signaling pathway mediated by SK via GPER in SKOV3 and A2780 cells. (a) The expression of EGFR, p-EGFR, PI3K, AKT and p-AKT following G-1 and G-15 treatment together with 8.6 *μ*M SK in SKOV3 cells. (b) The expression of EGFR, p-EGFR, PI3K, AKT, and p-AKT following G-1 and G-15 treatment together with 0.56 *μ*M SK in A2780 cells. The results are means of three independent replicates ± S.D.^*∗∗*^*P* < 0.01 or ^*∗*^*P* < 0.05 vs. control group, ^△△^*P* < 0.01 or ^△^*P* < 0.05 vs. SK treatment group were considered as statistically significant.

We then verified whether SK affected the expression of downstream genes through GPER. The choice of GPER agonists G1 and G15 was used to process SK. The above experiments showed that the IC50 of SK acting on SKOV3 and A2780 cells after 24 h was 8.6 *μ*M and 0.56 *μ*M; therefore, we used 10 nM G1 or 20 nM G15 combined with 8.6 *μ*M SK for SKOV3 cells and 0.56 *μ*M SK for A2780 cells for 24 h to observe the expression of EGFR, p-EGFR, PI3K, AKT, and p-AKT. The results showed that, in SKOV3 cells, G1 could upregulate the expression of EGFR, p-EGFR, PI3K, and p-AKT. G15 could downregulate the expression of p-EGFR and p-AKT, but the expression of EGFR and PI3K had no obvious effect. G1 cooperated with SK to downregulate the expression of EGFR, p-EGFR, PI3K, and p-AKT. Although G15 could not reverse the effect of SK on the expression of EGFR, it showed a clear reversal trend (Figure 5(a)). In A2780 cells, G1 could increase the expression of EGFR, p-EGFR, PI3K, and p-AKT protein expression, and G15 reduced expression of EGFR, p-EGFR, PI3K, and p-AKT protein expression. G1 also improved the effect of SK. However, G15 treatment reversed this trend (Figure 5(b)).

### 3.6. SK Inhibits SKOV3 and a2780 Cells Proliferation and Promotes Apoptosis Viability through a GPER-Mediated Manner

**Figure 6:**
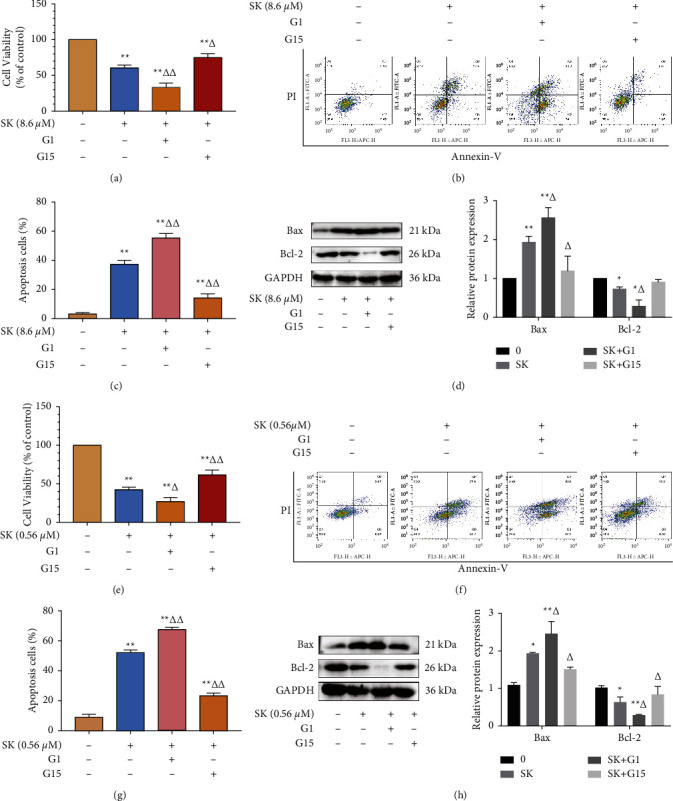
SK inhibits SKOV3 and A2780 cells viability and promotes apoptosis via the GPER mediated pathway. (a) Cell viability of SKOV3 cells treated with 8.6 *μ*M SK and 10 nM G1 or 20 nM G15 for 24 h. (b) Representative dot‑plots from cytometrically illustrating apoptotic status in SKOV3 cells treated with 8.6 *μ*M SK and 10 nM G1 or 20 nM G15 for 24 h. (c) Statistical analysis of apoptotic (percentage of cells in Q2 and Q3) SKOV3 cells. (d) The expression and quantitatively analysis of Bax and Bcl-2 proteins in SKOV3 cells treated with 8.6 *μ*M SK and 10 nM G1 or 20 nM G15 for 24 h. (e) Cell viability of A2780 cells treated with 0.56 *μ*M SK and 10 nM G1 or 20 nM G15 for 24 h. (f) Representative dot‑plots from cytometrically illustrating apoptotic status in A2780 cells treated with 0.56 *μ*M SK and 10 nM G1 or 20 nM G15 for 24 h. (g) Statistical analysis of apoptotic (percentage of cells in Q2 and Q3) A2780 cells. (h) The expression and quantitative analysis of Bax and Bcl-2 proteins in A2780 cells treated with 0.56 *μ*M SK and 10 nM G1 or 20 nM G15 for 24 h. The results are means of three independent replicates ± S.D. ^*∗∗*^*P* < 0.01 or ^*∗*^*P* < 0.05 vs. control group, ^△△^*P* < 0.01 or ^△^*P* < 0.05 vs. SK treating group were considered as statistically significant.

To further prove that the function of SK in inhibiting SKOV3 and A2780 cell proliferation and that the promotion of cell apoptosis may be mediated by GPER, we used 10 nM G1 or 20 nM G15 in combination with 8.6 *μ*M SK and 0.56 *μ*M SK with SKOV3 and A2780 cells, respectively. CCK-8 was used to detect cell viability, and the Annexin V-FITC/PI double staining assay was used to verify SKOV3 and A2780 cell apoptosis. Western blotting verified the expression of the apoptosis-related proteins Bax and Bcl-2. As expected, for SKOV3 cells, the viability was significantly lower in the SK + *G*1 group than in the SK + *G*15 group (Figure 6(a)). The apoptotic rate was increased in the SK + *G*1 group, and SK + *G*15 rescued apoptosis (Figures 6(b) and 6(c)). G1 stimulates the expression of Bax and inhibits the expression of Bcl-2. G15 reversed the expression of Bax but did not significantly change the expression of Bcl-2 (Figure 6(d)). Similar to the results for A2780 cells, G15 reversed the expression of Bcl-2 in the SK + *G*15 group (Figures 6(e)–6(h)).

### 3.7. SK Suppresses Tumor Growth through a GPER Mediated In Vivo

**Figure 7:**
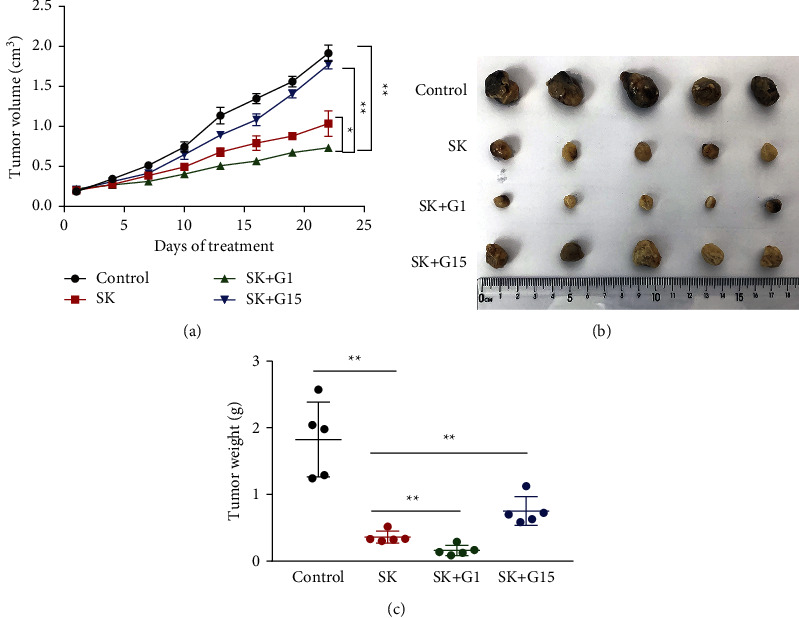
SK suppresses tumor growth via the GPER mediated pathway. (a) Mice were treated with 10 mg/kg SK and 1 *μ*g G1 or 5 *μ*g G15 for 22 days, tumor volume was measured every 3 days (*n* = 5). (b, c) After treatment with 10 mg/kg SK combine with 1 *μ*g G1 or 5 *μ*g G15 for 22 days, tumors in these groups were removed, tumor pictures were captured (b), tumors were weighed (c). ^*∗∗*^*P* < 0.01 or ^*∗*^*P* < 0.05 vs. control group.

To investigate whether the antitumor effect of SK also through GPER pathway in vivo, SKOV3 xenograft model was employed. After treatment with SK, SK + *G*1, SK + *G*15 for 22 days, the results showed that SK also possessed obvious antitumor effect in vivo, and G1 could increase the antitumor effect of SK. However, the antitumor effect of SK was attenuated by G15 to a great extent (Figures 7(a)–7(c)).

Our present study employed SKOV3, A2780 ovarian cancer cells, and xenograft model in vitro and in vivo to test the anticancer effect of SK and identified a novel mechanism by which SK induced apoptosis via GPER-mediated EGFR/PI3K/AKT downregulation. This might provide critical insights into the application of SK in ovarian cancer intervention.

## 4. Discussion

SK is the main component of Zicao, a Chinese herbal medicine with antitumor activity [12]. Although several studies have shown that SK induces apoptosis, necrosis, necroptosis, and migration by regulating many signaling pathways and molecular targets in various cancer cells [4], there are still few in-depth studies on ovarian cancer that need to be further explored. The most common histological subtype of all types of ovarian cancer is high-grade serous adenocarcinoma, which has the worst prognosis [13]. In this study, we used the high-grade serous adenocarcinoma SKOV3 and A2780 cell lines as models to study the effect of SK on ovarian cancer. Consistent with the results of previous studies, our results show that SK can inhibit malignant growth, increase the levels of LDH activity, and promote apoptosis of SKOV3 and A2780 cells [14, 15]. Mitochondria and cytochrome C are the key electron carriers in the electron transport chain and play a vital role in apoptosis induced by various stimuli. During apoptosis, *δψ*m is destroyed, causing cytochrome C to leak from the mitochondria into the cytosol. This process is regulated by the prosurvival/antiapoptotic protein Bcl-2 and the proapoptotic protein Bax, which leads to the activation and heptamerization of the adapter molecule apoptosis protease activator 1 (Apaf-1) to form apoptotic body complexes [16]. Therefore, Bcl-2 levels decreased, and Bax levels increased following SK treatment. We then established the relationship between active components, targets, pathways, biological processes, and diseases to explore the potential targets of Zicao in ovarian cancer through network pharmacology. The screening results showed that acetylshikonin, 5,8-dihydroxy-2-[(1R)-1-hydroxy-4-methylpent-3-en-1-yl]aphthalene-1,4-dione, isoarnebin 4, and 1-methoxyacetylshikonin may be effective ingredients in the treatment of ovarian cancer. It is well known that one of the effective ingredients, 5,8-dihydroxy-2-[(1R)-1-hydroxy-4-methylpent-3-en-1-yl]aphthalene-1,4-dione, is the chemical name of SK [17], which is consistent with the conclusions of previous studies. GO enrichment analysis revealed that membrane rafts, membrane microdomains, and membrane regions are the main cellular components, indicating that the function of Zicao in ovarian cancer is mainly through the cell membrane. KEGG pathway annotation indicated that the estrogen signaling pathway is the main mechanism of action of Zicao in the treatment of ovarian cancer. Modern pharmacological studies have shown that drug activity is closely related to the affinity and permeability of cell membranes. An important step in the action of traditional Chinese medicine is the binding of active components to cell membranes, specific enzymes, or cell receptors [18]. GPER, a seven-transmembrane protein, has been shown to play an intermediary role in rapid nongenomic signal transduction in response to estrogen [19]. We speculate that GPER may be involved in this biological process.

**Figure 8:**
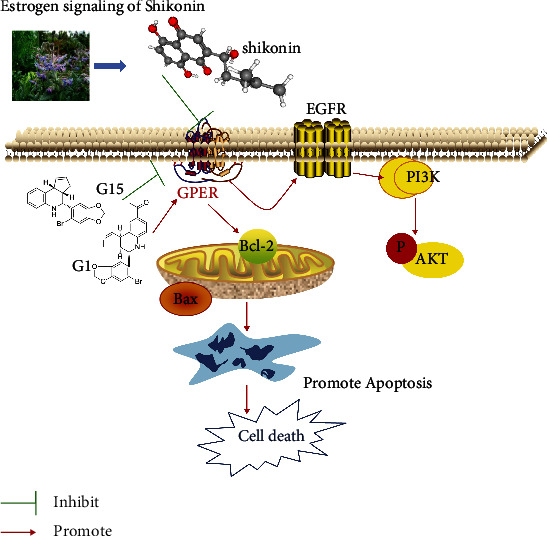
The potential mechanisms of SK.

Our results suggest that SK may induce cell proliferation inhibition and apoptosis through GPER in a dose-dependent manner. Studies have shown that GPER mediates nongenomic signal transduction of estrogen in a variety of estrogen-sensitive cancer cells by activating the nongenomic EGFR-dependent signaling pathway formed by PI3K/Akt [20, 21]. GPER leads to the activation of Src-related tyrosine kinases (Src) and matrix metalloproteinase (MMP) through the G*βγ* subunit, resulting in the release of epidermal growth factor (EGF) from the cell surface heparin-bound epidermal growth factor (HB-EGF) and EGF activation of EGFR [22]. EGFR, known as ERBB1, is a member of the ErbB (also known as HER) family and consists mainly of tyrosine kinases in the extracellular domain, transmembrane region, and cytoplasm. EGFR has been shown to be overexpressed in ovarian cancer cells. When binding to ligands, the formation of homopolymerization or heteropolymerization of EGFR with other members of the ErbB family leads to the phosphorylation of specific tyrosine residues in its cytoplasmic tail and then initiates a variety of downstream intracellular PI3K/AKT signaling pathways to promote malignant behavior of ovarian cells [23]. Previous evidence has shown that SK and its derivatives significantly inhibit the phosphorylation of PI3K and AKT in breast, cervical, gastric, lung, and colorectal cancer cells [24–28]. According to reports, p-AKT signaling is more active in ovarian cancer cells, and targeting PI3K/AKT signaling may be considered the main strategy for cancer treatment. Studies have confirmed that the antitumor effect of SK on MCF-7 and SK-BR-3 cells may be related to the inhibition of ER*α* and GPER downregulating EGFR/*β*-ERK [29]. Whether SK acts on the EGFR/PI3K/AKT transduction pathway through GPER binding is unclear. In the present study, we used GPER selective agonist G1 and antagonist G15 to determine the specificity of signal molecules in mediating the response of SK to ovarian cancer SKOV3 and A2780 cells. First, we found that SK inhibited the expression of EGFR, *p*-EGFR, PI3K, and *p*-AKT in SKOV3 cells in a concentration-dependent manner, indicating that SK needs EGFR, PI3K, and AKT for pharmacological action. Furthermore, *G*1 + SK inhibited the expression of EGFR and p-AKT. In contrast, G15 + SK eliminated the inhibitory effects on EGFR and *p*-AKT. In order to clarify the relationship between the inhibitory effect of SK on the proliferation and apoptosis of SKOV3 and A2780 cells and GPER, we found that SK combined with specific agonist G1 cell apoptosis was more obvious, and using the specific inhibitor G15 could reduce cell death. In addition, these findings were also be verified in SKOV3 ovarian cancer cells xenograft model. In vivo, G1 was able to increase the antitumor effect of SK; however, G15 could decrease this antitumor effect, which further proves that the function of SK is mediated by the GPER. The potential mechanisms of SK-induced necrosis and apoptosis in ovarian cancer cells are summarized in Figure 8. These results suggest that GPER may be an important gene in ovarian cancer cells proliferation inhibition and apoptosis induced by SK.

## 5. Conclusions

This study established the relationship between SK as an effective target for the treatment of ovarian cancer. The preliminary results show that SK may play a role through the estrogen signal GPER and its downstream EGFR/PI3K/AKT pathway in the treatment of ovarian cancer and verified that GPER is the key pathway. These findings provide a new direction for further study of the mechanism of SK in ovarian cancer.

## Figures and Tables

**Table 1:** Information for effective ingredients in Zicao.

MOL ID	Molecule Name	OB (%)	DL
MOL001494	Mandenol	42	0.19
MOL002372	(6Z,10E,14E,18E)-2,6,10,15,19,23-hexamethyltetracosa-2,6,10,14,18,22-hexaene	33.55	0.42
MOL002883	Ethyl oleate (NF)	32.4	0.19
MOL000359	sitosterol	36.91	0.75
MOL007714	1-methoxyacetylshikonin	73.09	0.29
MOL007715	5,8-dihydroxy-2-[(1R)-1-hydroxy-4-methylpent-3-en-1-yl]aphthalene-1,4-dione	54.64	0.29
MOL007716	acetylshikonin	62.39	0.27
MOL007722	Isoarnebin 4	64.79	0.2
MOL007728	lithospermidin A	75.08	0.38
MOL007734	5-[(E)-5-(3-furyl)-2-methyl-pent-2-enyl]-2,3-dimethoxy-p-benzoquinone	61.8	0.24
MOL007735	Des-O-methyllasiodiplodin	30.12	0.20
MOL007736	Lithospermidin B	60.48	0.39

## Data Availability

All data of this study are reflected in the original manuscript.
